# Training needs and service capacity gaps among primary healthcare workers in Henan Province, China: a large cross-sectional study

**DOI:** 10.1186/s12875-026-03348-9

**Published:** 2026-05-11

**Authors:** Yaping Tian, Nengguang Dai, Qiuping Zhao, Mingyang Yu, Hongwei Wen, Rongmei Liu

**Affiliations:** 1 Department of Health management, Zhengzhou Health College, Zhengzhou, Henan Province 450052 China; 2 Grassroots Health and Health Department, Health Commission of Henan Province, Zhengzhou, Henan Province 450046 China; 3 Henan Key Laboratory for Health Management of Chronic Diseases, Central China Fuwai Hospital, Central China Fuwai Hospital of Zhengzhou University, Zhengzhou, Henan Province 450003 China

**Keywords:** Primary healthcare, Training needs, Workforce development, Service capacity, China

## Abstract

**Background:**

Primary care training is a key strategy for strengthening healthcare services in China. This study aimed to systematically assess the training needs and service capacity improvement demands among primary healthcare workers in Henan Province, and to examine regional and role-specific variations, providing evidence for optimizing primary care workforce development.

**Methods:**

A cross-sectional online survey was conducted between April 11 and April 22, 2024, among 136,040 primary healthcare workers registered on the “Huayi Mobile Medical Education” training platform across 18 cities in Henan Province. A self-designed questionnaire assessed demographic characteristics, training needs, and service capacity enhancement. Descriptive statistics and chi-square tests were used to explore differences across regions and professional roles. Multivariable logistic regression models were constructed to examine associations between professional role and high training demand for selected core competencies, adjusting for institution type, professional title, and job position.

**Results:**

Overall, primary healthcare workers reported high demand (>75%) for training in the diagnosis and treatment of common diseases, rational drug use, and emergency response skills. Significant regional differences were observed, with certain areas showing higher demand for training in Traditional Chinese Medicine techniques, infectious disease control, or rehabilitation services. Training needs also varied by professional role: clinical staff emphasized clinical knowledge and Traditional Chinese Medicine, public health workers prioritized essential public health services, and nursing and technical personnel focused on first aid and emergency response.

**Conclusions:**

Training needs among primary healthcare workers in Henan Province are urgent and diverse, with clear regional and role-specific variations. These findings highlight the need for a competency-oriented, context-specific training system that improves relevance and practical applicability, thereby enhancing primary care service capacity and supporting the efficient allocation of health resources.

**Supplementary Information:**

The online version contains supplementary material available at 10.1186/s12875-026-03348-9.

## Background

Within the global healthcare system, primary care plays a pivotal role as the first line of defense in safeguarding public health [1, 2]. As a developing country with a large population, the improvement and development of China’s primary healthcare service system hold profound significance for enhancing the health level of the entire population, promoting social equity, and alleviating issues such as the uneven distribution of medical resources [3, 4]. Primary care services are directly oriented toward urban and rural residents, providing comprehensive basic medical services including diagnosis and treatment of common diseases, preventive care, management of chronic disorders, and health education [5, 6]. These services play an irreplaceable role in disease prevention, early diagnosis, and health promotion [7, 8].

With China’s rapid economic development and comprehensive social progress, public demand for healthcare services is increasingly growing and diversifying [9]. Concurrently, the accelerated aging of the population, the increasing burden of chronic diseases, and the rising health awareness among residents have imposed higher requirements on the quality and efficiency of primary healthcare services [10–12]. However, deficiencies remain in the professional competence, service capabilities, and knowledge structure of China’s primary healthcare workforce, which hinder the ability to meet the growing demand for primary care services [13, 14]. As direct workers of primary care, the professional proficiency and service capacity of primary healthcare workers directly influence the quality and effectiveness of primary care services [15–17].

In recent years, the Chinese government has continuously increased investment and actively promoted the development and reform of primary healthcare institutions [18]. As a result, the primary healthcare service system has been continuously improved, and service accessibility has been significantly enhanced [19]. However, the training system for primary healthcare workers in China remains insufficiently developed. Previous studies have reported that training content is often poorly aligned with the practical needs of primary healthcare workers. For example, qualitative research in township health centres found that some training programs focused on advanced clinical techniques that were rarely applicable in primary care settings, while other programs repeated similar topics without addressing daily service demands. In addition, short training duration and limited opportunities for hands-on practice were frequently reported as barriers to effective learning [20].

Evidence also suggests that the demand for professional development among primary healthcare workers is substantial. A systematic review including 38 cross-sectional studies involving 35,545 village doctors reported extensive training needs, particularly in the diagnosis and treatment of common diseases and public health management [21].

These issues severely restrict the enhancement of professional competence among primary healthcare workers and the quality of primary care services. Relevant studies indicate that effective training can significantly improve the professional knowledge and skills of primary healthcare workers, strengthen their ability to respond to complex medical situations, and thereby enhance patient satisfaction and the overall quality of primary care services [4–6, 10]. In recent years, increasing attention has been paid to the training and capacity development of primary healthcare workers in China. Previous studies have explored continuing medical education and competency development among village doctors and primary healthcare workers, and have reported considerable demand for training in areas such as common disease management and public health services [21]. However, most existing studies have focused on specific professional groups or limited geographic areas. Large-scale empirical studies that simultaneously examine training needs and broader service capacity improvement demands among diverse primary healthcare workers remain limited.

As one of the most populous provinces in China with substantial urban–rural disparities and a large primary healthcare workforce, Henan represents a typical central Chinese province undergoing primary care reform. This makes it a meaningful context for examining workforce development challenges. Therefore, this study selected Henan Province as the research site to reflect the current status of training in primary healthcare institutions, analyze the service capacity and training needs of primary healthcare workers in China, and provide empirical support for improving the quality of primary healthcare services and optimizing the allocation of human resources in primary care.

## Methods

### Study design and participants

From April 11 to 22, 2024, a cross-sectional survey was conducted among healthcare workers at primary healthcare institutions across 18 cities in Henan Province via the “Huayi Mobile Medical Education” comprehensive training platform. The study subjects were registered users of this platform. (In 2024, the platform had nearly 170,000 registered users, of whom approximately 165,900 had completed online training. The respondents in this survey accounted for approximately 80% of the total user base. The platform covers township health centers, county (district)-level hospitals, village clinics, and community health service centers in all cities and counties of Henan Province, and is used for online training and annual competency assessment of primary healthcare workers.) A total of 136,511 questionnaires were initially collected. After excluding questionnaires with incorrect address information or non-compliant IP address records, 136,040 valid questionnaires were retained for final analysis.

### Questionnaire development

The questionnaire was developed based on a review of relevant literature and expert consultation. An expert panel consisting of eight specialists in primary healthcare management, general practice, public health, and health services research was invited to review the initial questionnaire framework. All experts had extensive experience in primary healthcare policy or practice. The experts evaluated the relevance, clarity, and completeness of the questionnaire items and provided suggestions for modification. The research team revised the questionnaire accordingly. Frontline primary healthcare workers were not included in the expert consultation stage because the purpose of the consultation was to refine the questionnaire framework from a policy and research perspective. However, frontline workers were the target respondents of the main survey. Prior to the formal survey, a pilot test was conducted among a small group of primary healthcare workers to assess the clarity and feasibility of the questionnaire. Minor wording adjustments were made based on their feedback, and the pilot participants were not included in the final analysis.

The final questionnaire comprised three sections: (1) demographic and professional characteristics; (2) perceived training needs across multiple domains of primary care practice; and (3) perceived needs for primary care service capacity improvement. Training needs = perceived gaps in knowledge/skills requiring formal education. Service capacity improvement demands = perceived need to enhance ability to deliver services (organizational + technical). Training needs represent one pathway to improving service capacity, but service capacity also includes systemic and structural factors. All items were presented as single- or multiple-choice questions, allowing respondents to select all options that reflected their perceived needs.

The questionnaire was not intended to function as a psychometric scale measuring latent constructs, but rather as a policy-oriented needs assessment tool designed to capture the breadth of perceived training demands across a large and heterogeneous primary healthcare workforce. Given this descriptive purpose, formal psychometric testing (e.g., construct validity or internal consistency) was not conducted.

### Position division of health service personnel in primary medical institutions

Based on relevant policy documents and management regulations for primary healthcare institutions in Henan Province, the classification of job positions in this paper primarily relies on the following four core dimensions: First, the system of professional qualifications and title evaluation, which is based on documents such as the “Henan Province Criteria for Application and Evaluation of Senior Professional Titles in the Primary Healthcare Series”, categorizing positions according to legally defined practice categories and professional series, including clinical medicine, traditional Chinese medicine (TCM), public health, dentistry, nursing, pharmacy, and laboratory medicine. Second, the functional positioning of institutions and departmental setup, which, in accordance with standards such as the “Basic Standards for Rehabilitation Medicine Departments in Township Health Centers” and the “Requirements for Family Doctor Contracted Services and Public Health Department Setup”, clarifies the staffing standards and functional divisions for positions like general practice, public health, rehabilitation, and medical technology. Third, service models and team collaboration needs, where within the framework of integrated medical and preventive care and family doctor contracted services, general practitioners serve as the core, integrating public health personnel, nursing staff, and others to form collaborative teams. This approach adheres to the nationally unified standards for health professional and technical qualifications while fully incorporating the actual functional positioning and service innovation needs of primary healthcare institutions in Henan Province.

### Data analysis

This study employed a research design combining descriptive analysis with a policy-oriented approach, deliberately limiting the analytical scope to descriptive statistics and exploratory comparisons. The aim was to elucidate the overall patterns of training needs across different regions and professional roles. Descriptive statistics were used to summarize participants’ demographic characteristics, the distribution of perceived training needs, and the demands for service capacity enhancement. Categorical variables were presented as frequencies and percentages. The Chi-square (*χ*²) test was employed to explore the differential patterns in reported training needs between different regions and professional roles. The significance level was set at *α* = 0.05.

In addition to descriptive analysis, multivariate logistic regression analysis was conducted for selected core training areas to investigate whether professional roles and institutional context were independently associated with a high perceived training need. The dependent variables were the scores derived from the corresponding questionnaire dimensions. Independent variables included respondents’ professional and institutional characteristics, such as job category, type of primary healthcare institution, and regional distribution. Categorical variables were entered into the regression models using dummy variable coding. Although working years was initially collected in the questionnaire, this variable was excluded from the regression analysis due to substantial inconsistencies and missing values in the responses.

## Results

### Basic characteristics of respondents

A total of 136,040 valid questionnaires were included in the analysis, representing primary healthcare workers from diverse regions, institution types, professional titles, and job positions (Table 1). The largest proportions of respondents were from Nanyang, Zhoukou, Luoyang, and Anyang. Nearly half of the participants worked in village clinics, followed by those from township health centers. Most respondents held junior professional titles, while senior titles were relatively rare. In terms of job positions, over half of the participants were clinical physicians, including general practitioners and practitioners of TCM.

**Table 1:** Basic information of primary healthcare workers

Basic Information	Category	Number of people	Percentage (%)
Regions	ZhengZhou	9872	7.3
	KaiFeng	7316	5.4
	LuoYang	11,221	8.2
	PingDingShan	5776	4.2
	AnYang	11,193	8.2
	HeBi	2249	1.7
	XinXiang	9363	6.9
	JiaoZuo	4597	3.4
	PuYang	5113	3.8
	XuChang	4155	3.1
	LuoHe	3888	2.9
	SanMenXia	3440	2.5
	NanYang	19,179	14.1
	ShangQiu	9626	7.1
	XinYang	5414	4.0
	ZhouKou	11,681	8.6
	ZhuMaDian	10,929	8.0
	JiYuan	1028	0.8
Primary Healthcare Institutions	Village Clinics	62,743	46.1
	Community Health Service Centers	16,830	12.4
	Township Health Centers	53,714	39.5
	Health Management Institutions	654	0.5
	Others	2099	1.5
Professional Technical Title	Unclassified Title	38,729	28.5
	Junior Title	65,691	48.3
	Intermediate Title	28,235	20.8
	Associate Senior Title	3098	2.3
	Full Senior Title	287	0.2
Job Position	General Practice, Traditional Chinese Medicine, or Other Clinical Physicians	70,212	51.6
	Public Health or Preventive Care	26,970	19.8
	Nursing	19,352	14.2
	Rehabilitation	1528	1.1
	Medical Technology	12,486	9.2
	Administration	2670	2.0
	Others	2822	2.1

### Overall patterns of training needs and service capacity improvement demands in Henan Province

**Fig. 1:**
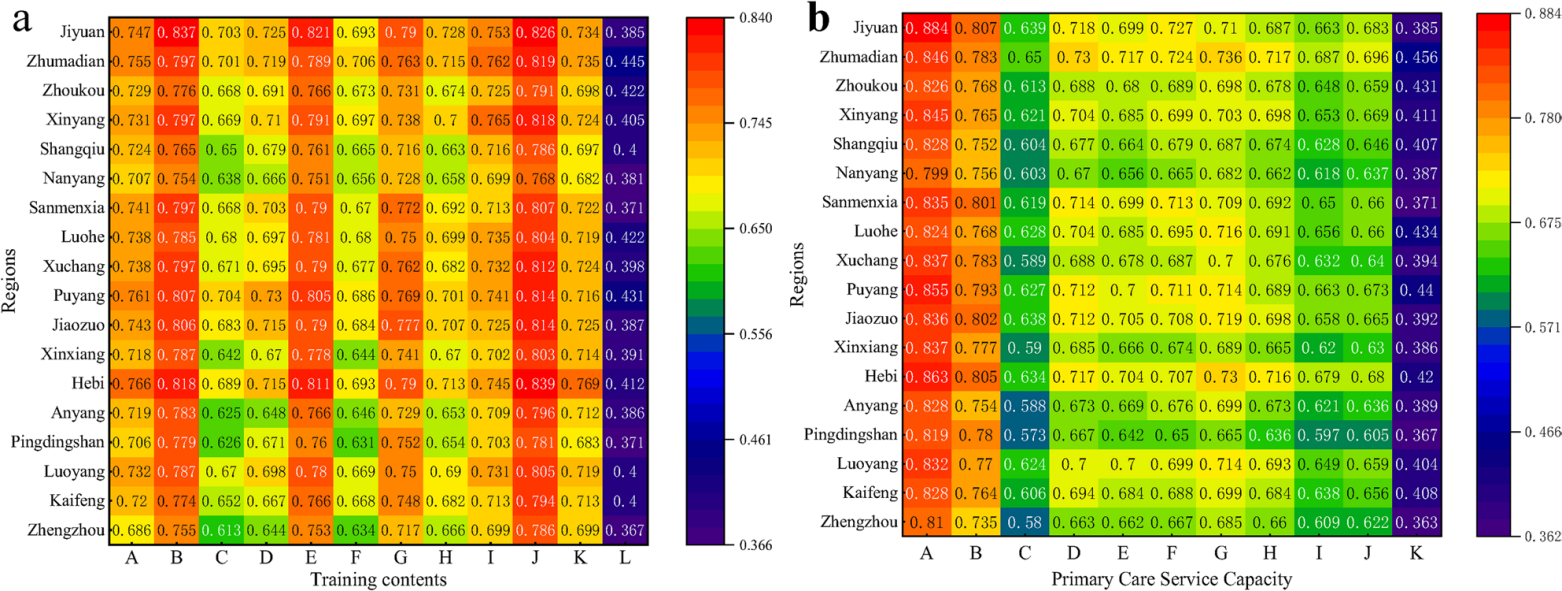
Training content and service capacity improvement needs among primary healthcare workers across different regions in Henan Province. **a** Distribution of training content needs across regions. **b** Distribution of perceived needs for primary healthcare service capacity improvement across regions. Training content categories include: A, Comprehensive general practice services; B, Diagnosis and treatment of common diseases; C, Basic public health services; D, Chronic disease management; E, Rational drug use in primary care; F, Professional ethics and health regulations; G, Appropriate TCM techniques; H, Community-based healthcare, rehabilitation, and nursing; I, Prevention and control of emerging infectious diseases; J, Emergency and first-aid techniques; K, Interpretation of auxiliary examination results; L, Others. Service capacity categories include: A, Clinical knowledge and skills; B, TCM knowledge and skills; C, Home-based care; D, Rehabilitation knowledge and skills; E, Nutrition and healthcare knowledge and skills; F, Health management knowledge and skills; G, Medical psychology and psychological counseling; H, Humanistic education and doctor–patient communication skills; I, Community management competence; J, Work regulations and service standards; K, Others

Across Henan Province, primary healthcare workers reported consistently high demand for training in core clinical competencies, including the diagnosis and treatment of common diseases, rational drug use, and emergency and first-aid skills. In most regions, demand for these training areas exceeded 75% (Fig. 1a). In contrast, demand for training related to basic public health services, chronic disease management, professional ethics, and community-based rehabilitation and nursing was relatively lower in several areas, with reported levels generally below 70%. Regarding service capacity improvement, demand for enhanced clinical knowledge and skills was consistently high across the province, exceeding 80% in most regions (Fig. 1b). Demand for competencies related to TCM was also prominent in several areas, whereas demand for home-based nursing services was comparatively lower in some regions, generally below 60%.

### Regional variations in training needs and service capacity improvement demands

Although overall patterns were broadly similar across Henan Province, some regional variations were observed. For example, Hebi reported relatively higher demand for training in general practice concepts, appropriate TCM techniques, and interpretation of auxiliary examination results. In contrast, Zhumadian and Xinyang showed comparatively greater demand for training in the prevention and control of emerging infectious diseases. With respect to service capacity improvement, Zhumadian reported relatively higher demand for rehabilitation-related competencies and medical psychological counseling skills compared with other regions.

### Differences in training needs across job positions

Significant differences in training needs were observed across job positions (*χ*² tests, all *P* < 0.001). Clinical physicians, including general practitioners and practitioners of TCM, most frequently reported demand for training in the diagnosis and treatment of common diseases. Public health and preventive care personnel primarily expressed demand for training related to essential public health services. Nursing staff, medical technology personnel, and rehabilitation workers reported comparatively higher demand for training in emergency response, rehabilitation, and community-based care-related competencies, reflecting the functional focus of their respective roles. Regarding participation in remote learning, differences in daily learning duration were observed across job positions. However, across all job categories, more than 70% of respondents reported spending less than two hours per day on remote learning activities (Table 2).

**Table 2:** Comparison of training content and duration of remote learning among primary healthcare workers in different job positions

	General Practice, Traditional Chinese Medicine, or Other Clinical Physicians	Public Health or Preventive Care	Nursing	Rehabilitation	Medical Technology	Administration	Others	*χ* ^2^	*P*-value
Comprehensive General Practice Services	53,133(75.7)	19,242(71.3)	13,454(69.5)	1057(69.2)	8110(65)	1906(71.4)	1755(62.2)	1328.84	< 0.001
Diagnosis and Treatment of Common Diseases	58,361(83.1)	20,385(75.6)	13,778(71.2)	1130(74)	8697(69.7)	1944(72.8)	1851(65.6)	2893.08	< 0.001
Basic Public Health Services	44,688(63.6)	21,081(78.2)	12,102(62.5)	881(57.7)	6932(55.5)	1905(71.3)	1668(59.1)	3080.36	< 0.001
Chronic Disease Management	48,639(69.3)	20,135(74.7)	12,531(64.8)	940(61.5)	7106(56.9)	1911(71.6)	1670(59.2)	1996.74	< 0.001
Rational Drug Use in Primary Care	57,346(81.7)	20,429(75.7)	13,843(71.5)	1060(69.4)	8510(68.2)	1992(74.6)	1891(67)	2645.19	< 0.001
Professional Ethics and Health Regulations	46,375(66)	18,542(68.8)	12,918(66.8)	990(64.8)	7985(64)	1955(73.2)	1797(63.7)	302.78	< 0.001
Appropriate Traditional Chinese Medicine Techniques	55,585(79.2)	19,905(73.8)	13,025(67.3)	1204(78.8)	7554(60.5)	1975(74)	1734(61.4)	3472.55	< 0.001
Community-Based Healthcare, Rehabilitation, and Nursing	46,875(66.8)	19,343(71.7)	13,808(71.4)	1189(77.8)	7422(59.4)	1898(71.1)	1715(60.8)	1208.47	< 0.001
Prevention and Control of Emerging Infectious Diseases	51,415(73.2)	19,803(73.4)	13,702(70.8)	1018(66.6)	8344(66.8)	1888(70.7)	1867(66.2)	719.95	< 0.001
Emergency and First-Aid Techniques	58,126(82.8)	20,885(77.4)	15,059(77.8)	1143(74.8)	9135(73.2)	2052(76.9)	1972(69.9)	1648.81	< 0.001
Interpretation of Auxiliary Examination Results	51,716(73.7)	18,650(69.2)	12,743(65.8)	1062(69.5)	8682(69.5)	1856(69.5)	1731(61.3)	1014.12	< 0.001
Others	28,599(40.7)	11,004(40.8)	7317(37.8)	594(38.9)	4452(35.7)	1062(39.8)	1152(40.8)	253.21	< 0.001
Remote Learning Duration								566.94	< 0.001
Less than 2 h	51,351(73.1)	19,600(72.7)	14,293(73.9)	1072(70.2)	9096(72.8)	2105(78.8)	1978(70.1)		
2–4 h	16,325(23.3)	6304(23.4)	4200(21.7)	382(25)	2840(22.7)	444(16.6)	578(20.5)		
More than 4 h	1285(1.8)	597(2.2)	401(2.1)	48(3.1)	255(2)	49(1.8)	59(2.1)		
Others	1251(1.8)	469(1.7)	458(2.4)	26(1.7)	295(2.4)	72(2.7)	207(7.3)		

Values are presented as *n* (%), where percentages are shown in parentheses

### Differences in perceived service capacity improvement needs and learning interests across job positions

Role-specific differences were also observed in perceived needs for service capacity improvement (*χ*² tests, all *P* < 0.001). Clinical physicians most frequently expressed demand for enhanced clinical and TCM–related competencies. Nursing staff reported relatively greater demand for training in home-based care, whereas rehabilitation personnel primarily prioritized rehabilitation-related knowledge and skills. Management personnel consistently reported high demand for training in non-clinical competencies, including health management, communication, psychological counseling, and organizational governance. Regarding learning content preferences, standardized knowledge content was most commonly preferred across all job categories, followed by cutting-edge knowledge. Preferences differed significantly across job positions (*P* < 0.001; Table 3).

**Table 3:** Comparison of primary healthcare workers in different job positions regarding perceived needs for service capacity improvement and learning interests

	General Practice, Traditional Chinese Medicine, or Other Clinical Physicians	Public Health or Preventive Care	Nursing	Rehabilitation	Medical Technology	Administration	Others	*χ* ^2^	*P*-value
Clinical Knowledge and Skills	61,121(87.1)	21,334(79.1)	15,231(78.7)	1198(78.4)	9721(77.9)	2023(75.8)	1966(69.7)	2458.377	< 0.001
Traditional Chinese Medicine Knowledge and Skills	57,445(81.8)	20,514(76.1)	13,567(70.1)	1247(81.6)	7965(63.8)	1997(74.8)	1765(62.5)	3493.83	< 0.001
Home-Based Care	40,111(57.1)	17,519(65.0)	13,187(68.1)	957(62.6)	7581(60.7)	1805(67.6)	1645(58.3)	1230.99	< 0.001
Rehabilitation Knowledge and Skills	47,547(67.7)	19,260(71.4)	13,981(72.2)	1277(83.6)	8002(64.1)	1957(73.3)	1760(62.4)	853.57	< 0.001
Nutrition and Health Care Knowledge and Skills	46,152(65.7)	19,411(72)	13,739(71.0)	1041(68.1)	8208(65.7)	1931(72.3)	1787(63.3)	694.80	< 0.001
Health Management Knowledge and Skills	46,749(66.6)	19,805(73.4)	13,721(70.9)	1076(70.4)	8234(65.9)	1990(74.5)	1792(63.5)	771.17	< 0.001
Medical Psychology and Psychological Counseling	48,736(69.4)	19,147(71)	13,932(72)	1099(71.9)	8436(67.6)	1969(73.7)	1845(65.4)	299.60	< 0.001
Humanistic Education and Doctor–Patient Communication Skills	47,155(67.2)	18,583(68.9)	13,547(70)	1071(70.1)	8227(65.9)	1959(73.4)	1839(65.2)	237.48	< 0.001
Community Management Competence	43,832(62.4)	18,408(68.3)	12,627(65.2)	955(62.5)	7342(58.8)	1942(72.7)	1605(56.9)	744.27	< 0.001
Work Regulations and Service Standards	44,445(63.3)	18,386(68.2)	12,968(67)	1002(65.6)	7892(63.2)	1979(74.1)	1765(62.5)	463.52	< 0.001
Others	28,219(40.2)	11,242(41.7)	7850(40.6)	634(41.5)	4599(36.8)	1082(40.5)	1137(40.3)	200.42	< 0.001
Remote Learning Duration								656.74	< 0.001
Learning Content	11,827(16.8)	4230(15.7)	3265(16.9)	311(20.4)	2028(16.2)	526(19.7)	585(20.7)		
Standardized Knowledge Content	49,601(70.6)	18,900(70.1)	13,101(67.7)	1005(65.8)	8698(69.7)	1625(60.9)	1810(64.1)		
Cutting-Edge Knowledge Content	6593(9.4)	2912(10.8)	1866(9.6)	126(8.2)	1097(8.8)	394(14.8)	285(10.1)		
Less Common Knowledge Content	2191(3.1)	928(3.4)	1120(5.8)	86(5.6)	663(5.3)	125(4.7)	142(5)		

Values are presented as *n* (%), where percentages are shown in parentheses

### Regional variation in demand for clinical theoretical knowledge

**Fig. 2:**
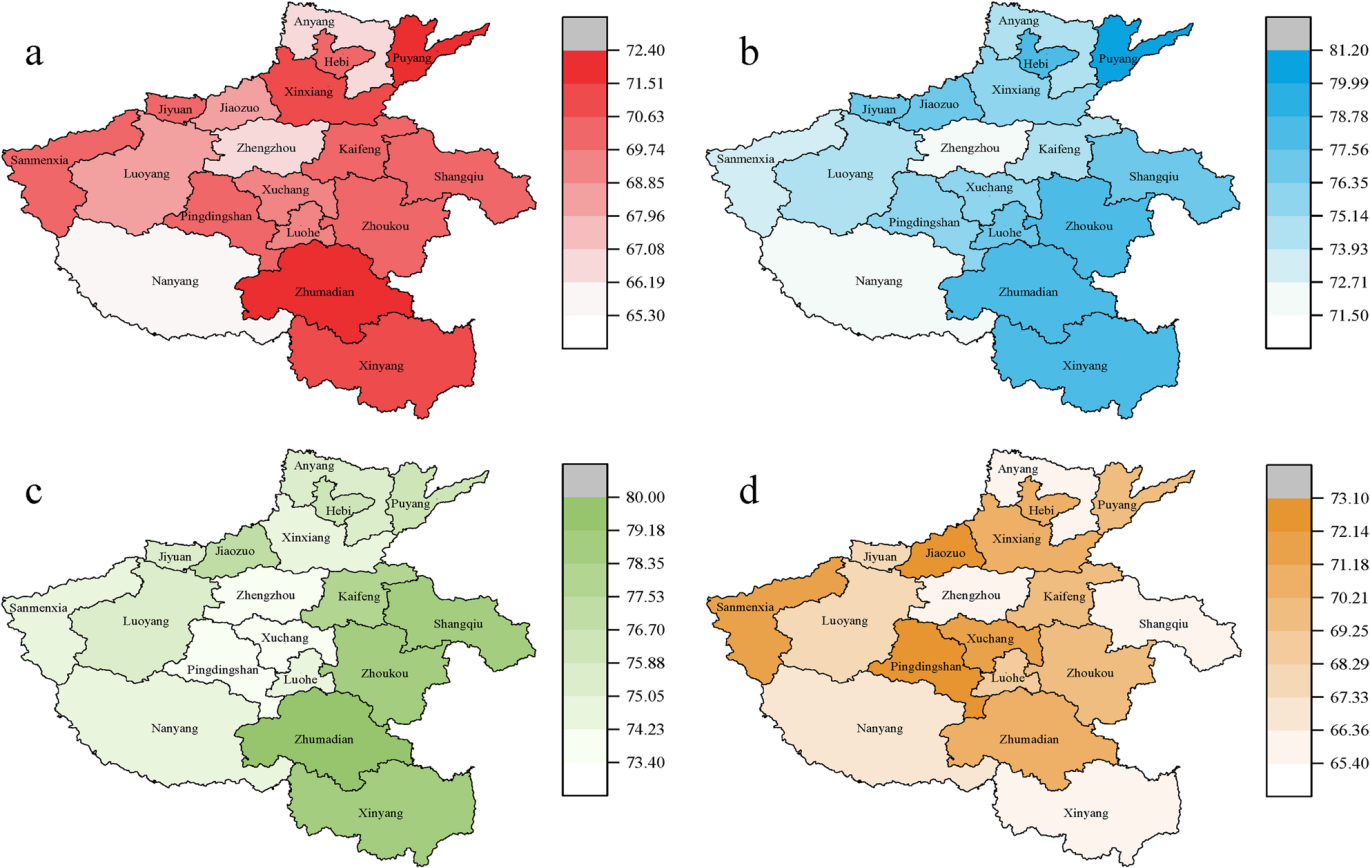
Regional variation in demand for clinical theoretical knowledge among primary healthcare workers in Henan Province. **a** Demand for training in basic pharmacological knowledge and rational drug use. **b** Demand for training in basic theoretical knowledge of general practice. **c** Demand for training in basic clinical medical theory. **d** Demand for training in basic theoretical knowledge of TCM

Spatial analysis further revealed regional variation in demand for specific knowledge domains. Demand for training in basic pharmacology and rational drug use was relatively higher in several regions(Fig. 2a), while demand for foundational clinical medicine and general practice theory remained consistently high across all regions, exceeding 70% (Fig. 2). In contrast, demand for training in fundamental TCM theory was more pronounced in selected regions compared with others.

### Independent association of professional roles with demand for training in common disease diagnosis and treatment

Multivariate logistic regression analysis showed that, after adjusting for institution type, professional title, and job position, professional role remained independently associated with high training demand. No significant differences were observed among medical technicians, rehabilitation staff, and nurses compared with the reference group. Respondents from village clinics and township health centers showed higher demand, while those with junior professional titles were more likely to report high demand than those with associate senior titles. The overall findings were consistent with the descriptive analysis, further supporting the robustness of our results (Table S1).

## Discussion

This study provides a large-scale overview of training needs and perceived service capacity improvement demands among primary healthcare workers in Henan Province. The findings reveal consistently high demand for training in core clinical competencies, notable regional variations in specific training priorities, and role-specific differences across job positions. These results provide important insights for optimizing training strategies and strengthening the primary healthcare workforce.

First, the consistently high demand for training in core clinical competencies, including the diagnosis and treatment of common diseases, rational drug use, and emergency and first-aid skills, likely reflects not only their central role in delivering frontline clinical services but also the gap between current clinical capacity and the demands of real-world practice. Previous studies have shown that strengthening clinical competency and continuing professional education is critical for improving the quality of primary care services, particularly in resource-limited settings [22–24]. In many township health centers and village clinics, healthcare workers are responsible for managing a wide range of common conditions with limited access to specialist support. As a result, strengthening clinical competence remains a fundamental requirement for improving the quality of primary care. Similar findings have been reported in recent studies examining training needs among primary healthcare workers in China [25, 26].

Second, the observed regional variations in training needs may be related to differences in local health service capacity, population health profiles, and resource distribution. For example, higher demand for training in emerging infectious disease prevention in Xinyang may reflect recent experiences with public health emergencies or local disease patterns. Similarly, the stronger demand for rehabilitation-related competencies in Zhumadian may be associated with population aging and the increasing prevalence of chronic diseases [27, 28]. These findings highlight the importance of adopting regionally tailored training strategies rather than relying solely on standardized national training programs.

Third, clear role-specific differences in training needs were identified across job positions. Clinical physicians primarily prioritized clinical diagnostic and treatment competencies, while public health personnel emphasized essential public health services. Nursing and rehabilitation staff demonstrated stronger demand for training related to community-based care, rehabilitation, and emergency response. Although such differences may appear intuitive, the present study provides large-scale empirical evidence quantifying the extent of these role-specific training priorities among primary healthcare workers. Previous studies have similarly highlighted the importance of profession-specific training strategies in strengthening healthcare workforce capacity [29–31]. These findings suggest that training programs should be designed with role-specific curricula to better match practical work demands.

The findings of this study are broadly consistent with previous international research indicating that strengthening clinical capacity and continuing professional education are key priorities for primary healthcare workforce development [32]. However, the Chinese primary healthcare system also has several distinctive features that shape training needs. In China, primary healthcare institutions such as township health centers and village clinics play a dual role in delivering both clinical services and essential public health programs, including chronic disease management and community-based preventive services [33]. In addition, the integration of TCM into primary healthcare practice is a unique characteristic of the Chinese healthcare system. The relatively high demand for TCM-related training observed in this study reflects the continued importance of TCM in community-based healthcare services, particularly in rural areas [25, 34].

Henan Province also has several contextual characteristics that may influence training needs. As one of the most populous provinces in China, Henan faces substantial demand for primary healthcare services, particularly in rural areas where healthcare resources remain relatively limited. Primary healthcare workers often serve large populations with constrained staffing and infrastructure, which may explain the strong demand for training aimed at improving practical clinical skills and service capacity. Similar workforce challenges have been documented in other studies examining rural health workforce development in China [35, 36].

Based on these findings, several policy implications can be considered. First, training programs for primary healthcare workers should prioritize strengthening core clinical competencies, particularly in the management of common diseases, rational drug use, and emergency care. Second, policymakers should consider adopting region-specific training strategies that address local health needs and resource conditions. Third, training programs should be designed with profession-specific modules, ensuring that the educational content aligns with the practical responsibilities of different healthcare roles. Finally, greater integration of TCM training within primary healthcare education may further support the development of comprehensive community-based healthcare services. Strengthening continuing education systems for primary healthcare workers has been widely recognized as a key strategy for improving primary healthcare capacity [16, 29, 30].

Overall, optimizing training systems for primary healthcare workers is essential for strengthening primary care service capacity. The findings of this study provide evidence that can inform the design of more targeted and context-specific training strategies in Henan Province and potentially in other regions with similar healthcare system characteristics.

### Limitations

Despite the strengths of the large sample size and broad geographic coverage, several limitations should be acknowledged. First, the study participants were registered users of the Huayi Mobile Medical Education platform. The study may have excluded elderly primary healthcare workers who rarely use online platforms, those in remote village clinics with limited informatization access, and those who did not register on the platform. Second, the study relied on self-reported questionnaire data, which may be subject to reporting bias. Third, the cross-sectional design limits the ability to establish causal relationships between training needs and influencing factors. Fourth, although the survey included a large number of primary healthcare workers across Henan Province, demographic variables such as age and gender were not collected, which limited further exploration of their potential influence on training needs. Finally, some subgroups contained relatively small sample sizes, which may affect the precision of subgroup comparisons. Future studies incorporating more detailed demographic information and longitudinal designs may provide deeper insights into the evolving training needs of the primary healthcare workforce.

## Conclusion

This study provides a comprehensive assessment of training needs and perceived service capacity improvement demands among primary healthcare workers in Henan Province. The findings indicate a consistently high demand for strengthening core clinical competencies, particularly in the diagnosis and treatment of common diseases, rational drug use, and emergency care. At the same time, notable regional and role-specific differences in training priorities were identified. These results highlight the importance of developing targeted and context-specific training strategies, including regionally adapted training programs and profession-specific curricula. Strengthening training systems for primary healthcare workers may contribute to improving service capacity and promoting the sustainable development of primary healthcare in China.

## Supplementary Information

Supplementary Material 1.

## Data Availability

The data supporting the conclusions of this study were derived from questionnaires administered during the research period. The datasets used and/or analyzed in the present study are available from the corresponding author upon reasonable request.

## Abbreviations

TCM: Traditional Chinese Medicine
a*OR*: Adjusted odds ratio
*CI*: Confidence interval
*SE*: Standard error
