# Supplementary material for: Training needs and service capacity gaps among primary healthcare workers in Henan Province, China: a large cross-sectional study
Source: BMC Prim Care. 2026 May 11;27:251. doi: 10.1186/s12875-026-03348-9 (PMC13330086; doi:10.1186/s12875-026-03348-9)
Supplement: Supplementary file 1 — Supplementary Material 1. [file 12875_2026_3348_MOESM1_ESM.docx]

Table S1. Multivariable logistic regression analysis of factors associated with high training demand for diagnosis and treatment of common diseases

| Variable | *β* | *SE* | *P-value* | a*OR* | 95%CI |
| --- | --- | --- | --- | --- | --- |
| Primary Healthcare Institutions |  |  |  |  |  |
| Village Clinics | 0.89 | 0.06 | <0.001 | 2.43 | 2.18-2.72 |
| Community Health Service Centers | 0.00 | 0.06 | 0.978 | 1.00 | 0.89-1.12 |
| Township Health Centers | 0.27 | 0.06 | <0.001 | 1.31 | 1.17-1.46 |
| Health Management Institutions | 0.21 | 0.11 | 0.066 | 1.23 | 0.99-1.53 |
| Professional Technical Title |  |  |  |  |  |
| Unclassified Title | 0.41 | 0.14 | 0.004 | 1.51 | 1.14-1.99 |
| Junior Title | 0.40 | 0.14 | 0.005 | 1.49 | 1.13-1.97 |
| Intermediate Title | 0.33 | 0.14 | 0.021 | 1.39 | 1.05-1.84 |
| Associate Senior Title | 0.02 | 0.15 | 0.88 | 1.02 | 0.77-1.37 |
| Job Position |  |  |  |  |  |
| General Practice, Traditional Chinese Medicine, or Other Clinical Physicians | 0.66 | 0.05 | <0.001 | 1.94 | 1.76-2.13 |
| Public Health or Preventive Care | 0.19 | 0.05 | <0.001 | 1.21 | 1.10-1.33 |
| Nursing | 0.10 | 0.05 | 0.051 | 1.10 | 1.00-1.21 |
| Rehabilitation | 0.00 | 0.08 | 0.987 | 1.00 | 0.86-1.16 |
| Medical Technology | 0.04 | 0.05 | 0.421 | 1.04 | 0.94-1.15 |
| Administration | 0.18 | 0.07 | 0.006 | 1.20 | 1.05-1.36 |
| Constant | 0.50 | 0.16 | 0.001 | 1.65 |  |

*β*: Regression coefficient. *SE*: Standard error. a*OR*: Adjusted odds ratio. *CI*: Confidence interval.
